# Two-year results after coronary stenting of small vessels in Japanese population using 2.25-mm diameter sirolimus-eluting stent with bioresorbable polymer: primary and long-term outcomes of CENTURY JSV study

**DOI:** 10.1007/s12928-018-0511-3

**Published:** 2018-01-18

**Authors:** Shigeru Saito, Kenji Ando, Yoshiaki Ito, Tetsuya Tobaru, Junji Yajima, Takeshi Kimura, Kazushige Kadota

**Affiliations:** 1 Department of Cardiology and Catheterization Laboratories, Shonan Kamakura General Hospital, Okamoto 1370-1, Kamakura, 247 8533 Japan; 2 Division of Cardiology, Kokura Memorial Hospital, Kokura, Japan; 3 Department of Cardiology, Saiseikai Yokohama City Eastern Hospital, Yokohama, Japan; 4 Department of Cardiology, Sakakibara Heart Institute, Tokyo, Japan; 5 Department of Cardiovascular Medicine, The Cardiovascular Institute, Tokyo, Japan; 6 Department of Cardiovascular Medicine, Graduate School of Medicine, Kyoto University, Kyoto, Japan; 7 Department of Cardiology, Kurashiki Central Hospital, Kurashiki, Japan

**Keywords:** Drug-eluting stent, Sirolimus, Bioresorbable, Very small vessel

## Abstract

Percutaneous coronary intervention (PCI) in coronary artery disease (CAD) with very small vessels remains challenging. The aim of this study is to evaluate the safety and effectiveness of the 2.25-mm diameter Ultimaster sirolimus-eluting stent in the treatment of Japanese patients with CAD due to lesions in very small vessels. The CENTURY JSV study is a prospective, multicentre, single-arm study. Seventy patients with lesions deemed suitable for implantation of a 2.25-mm diameter stent were enrolled at seven hospitals in Japan. Patients underwent clinical follow-up at 1-, 9-month, 1-, and 2-year after the PCI procedure. The primary endpoint was the major adverse cardiac event (MACE), a composite of cardiac death, target vessel myocardial infarction (MI), and clinically driven target lesion revascularization (TLR) free rate at 9-month following the procedure. The MACE-free rate was 97.1%, and the lower limit of the two-sided 95% confidence interval (CI) was 90.1%, which exceeded the threshold of 80% set as the performance goal. Angiographic in-stent and in-segment late loss at 9-month were 0.22 ± 0.31 and − 0.02 ± 0.34 mm, respectively. Between 9 months and 2 years, two additional TLRs occurred. Stent thrombosis, bleeding and vascular complication did not occur throughout 2 years. The 2.25-mm diameter Ultimaster^®^ bioresorbable-polymer sirolimus-eluting stent is safe and effective for treating lesions in very small coronary arteries throughout 2 years after stent implantation.

Clinical trial registration: UMIN000012928.

## Introduction

Over the history of percutaneous coronary intervention (PCI), in the era of plain old balloon angioplasty (POBA) and the following period in which bare metal stents (BMS) were used, clinical outcomes in small vessels were less favorable than those in large vessels and were far from satisfactory. Pfisterer et al. compared the outcomes between drug-eluting stents (DES) and BMS in the treatment of small and large vessels based on a 3-year analysis of the BASKET study [1], and reported that MACE was significantly more frequent with BMS than with DES in small vessels.

Only a few multicenter clinical studies have reported both clinical and angiographic outcomes for all enrolled patients at follow-up using stents smaller than 2.5 mm in diameter [2–7]. There are even fewer studies in which the second-generation DESs were used [3, 7], and there have been no studies that examined 2.25-mm diameter DESs with a bioresorbable polymer.

Asian population, including Japanese patients, are physically smaller than Western patients. In addition, about 40% of the Japanese patients undergoing PCI treatment are complicated by the presence of diabetes mellitus. Therefore, the coronary arteries to be treated are often narrower in Japanese patients than in their western counterparts. Consequently, considering that a higher percentage of small-diameter stents are used in such patients, it is important to verify the clinical outcomes associated with 2.25-mm diameter stents when establishing a treatment policy for PCI in Japanese patients.

Considering the situation described above, we conducted a multicenter clinical study of the 2.25-mm Ultimaster stent, which is a third-generation sirolimus-eluting stent (SES) with a bioresorbable polymer, in Japanese patients to evaluate the clinical and angiographic outcomes over a 9-month and longer follow-up period.

## Methods

### Study design and patient population

This study is a prospective, multicentre, single-arm study. The study was reviewed and approved by the Institutional Review Board of each site and conducted in compliance with the Declaration of Helsinki and Good Clinical Practice.

The inclusion/exclusion criteria were as follows.

Patients with ischemic heart disease due to significant stenosis in single lesions shorter than 38 mm (single stent for target lesion), and suitable for 2.25-mm diameter stent implantation were considered for enrollment.

Main exclusion criteria were renal failure requiring dialysis, acute myocardial infarction (AMI) within 48 h, bifurcation lesions requiring stenting of both main and side branch, ostial lesions, bypass grafts, left main trunk lesions, staged PCI, in-stent restenosis, and lesion requiring preparation other than balloon pre-dilation.

Prior to subject enrollment, patients received a full explanation of the study and provided written consent.

### Hypothesis

Bare metal stents less than 2.5 mm in diameter were not approved, and there was no DES less than 2.5 mm for which evaluation was defined in Japan at the start of the present study, due to the concern that they were associated with high rates of restenosis and a DES has just approved. Therefore, we designed the current study to evaluate the outcomes of patients treated with the 2.25-mm Ultimaster DES, and the results will be compared with historical control—POBA. Our hypothesis was that the lower limit of the two-sided 95% confidence interval (CI) of the MACE-free rate of 2.25-mm diameter Ultimaster stent exceeds 80%, which is the 9-month MACE-free rate of POBA based on previous study [8].

### Sample size calculation

Based on the outcomes reported in DES literature [9–11], assuming that the 9-month MACE-free rate of 2.25-mm diameter Ultimaster stent is 93% and the lower limit of the two-sided 95% CI to be at least 80%; to achieve 80% study power, 62 patients are required. Taking into account a dropout rate of 10%, we established 70 patients as the target sample size.

### Study device

The platform of the investigational device is a cobalt-chromium alloy 2-link, open-cell structure. Sirolimus, a drug inhibiting endothelial proliferation, is loaded at 3.9 μg/mm. The polymer is a poly (d, l-lactic acid)-poly (caprolactone) copolymer, which uses abluminal gradient coating technology. This polymer is designed to dissolve within 3–4 months, and the drug is designed to be gradually released for about 3 months.

The following 7 stent sizes were used in the present study: diameter: 2.25 mm, length: 12, 15, 18, 24, 28, 33, and 38 mm.

### Procedural and post-interventional practices

Only patients with a single lesion requiring treatment were enrolled. The use of a single stent was recommended except in case of bailout. Apart from mandatory lesion pre-dilatation, PCI was performed in accordance with the standard procedure of each site.

Antiplatelet therapy with aspirin and a P2Y12 inhibitor was started before the index procedure and dual antiplatelet therapy were maintained for 9 months following the procedure in all subjects.

### Follow-up, study endpoints, and definitions

Clinical follow-up was performed at 1, 9 months, 1, and 2 years. Serious adverse events were reported immediately at the time known by the investigators and other events were checked at each follow-up visit after the procedure. Angiographic follow-up was performed at 9-month follow-up.

The primary endpoint was the MACE-free rate at 9-month, and MACE was defined as; cardiac death, target vessel myocardial infarction (MI) [Q and non-Q wave], and clinically driven target lesion revascularization (TLR)-free rate. The secondary endpoints were: MACE-free rates at 1-year interval and the rates at 1-year interval thereafter until 5 years had elapsed from the time the procedure was performed, target lesion failure (TLF), target vessel failure (TVF), TLR, target vessel revascularization (TVR), cardiac death, and MI (Q- and non-Q-wave) were to be evaluated at 1, 9, 12 months, and yearly until 5 years (the definition of all endpoints is given in supplementary appendix). All endpoint-related events (death, MI, revascularization, stent thrombosis, bleeding, and vascular complication) were adjudicated by an independent Clinical Event Adjudication Committee.

Angiographic endpoints assessed at 9 months after PCI, both, in stent and in segment, were: acute gain, minimal lumen diameter (MLD), %diameter stenosis, late lumen loss (LLL), and binary restenosis rate.

Angiographic images were digitally recorded and assessed by Quantitative Angiographic Core Laboratory (Japan Cardio Core, Tokyo, Japan). Digital angiograms were analyzed offline by experienced personnel using an automated edge detection system (QCACMS, Medis Medical Imaging Systems, Nuenen, The Netherlands).

### Statistical analysis method

The main analysis in this study was to verify that the lower limit of exact two-sided 95% confidence intervals (CI) of the MACE-free rate at 9 months after PCI is more than 80% in the full analysis set (FAS). The point estimates and two-sided 95% CI using exact method were calculated for TLF-, TVF-, TLR-, and TVR-free rates. All the analyses were carried out using SAS Release 9.2 (SAS Institute Japan Ltd.).

## Results

### Baseline and procedural characteristics

A total of 70 patients were enrolled at 7 sites in Japan from April 16, 2014 to December 25, 2014. The mean age of the patients at the time of PCI was 70.4 ± 9.2 years; concomitant diseases including hypertension (87.1%), diabetes mellitus (37.1%), lower limb lesions (7.1%), and previous MI and PCI were present in 27.1 and 52.9% of the patients, respectively, reflecting the standard population of Japanese patients undergoing PCI (Table 1). In terms of lesion characteristics, as the target lesions were small-diameter vessels, most of them were located in the distal part of the left anterior descending artery and circumflex artery, and bifurcation lesions were slightly more prevalent (Table 2).

**Table 1:** Patient demographics

Number of patients	70
Age (mean ± SD)	70.4 ± 9.2
Gender, male (%)	77.1
Type of angina (%)	
Stable	87.1
Unstable	5.7
Silent ischemia	7.1
Diabetes (%)	37.1
IDDM (%)	0
Hypertension (%)	87.1
Dyslipidemia (%)	87.1
Cerebrovascular disease (%)	2.9
Peripheral artery disease (%)	7.1
Congestive heart failure (%)	4.3
Family history of CAD (%)	27.1
Current smoker (%)	11.4
Previous PCI (%)	52.9
Previous CABG (%)	0
Previous MI (%)	27.1
Previous Stroke (%)	11.4

**Table 2:** Lesion/procedural characteristics

Number of lesions	70
Target vessel location (%)	
LMT	0
LAD	32.9
LCx	42.9
RCA	24.3
Lesion classification (%)	
A	8.6
B1	22.9
B2	35.7
C	32.9
Bend (> 45°) (%)	8.6
Calcification^a^ (%)	7.1
Tortuosity^a^ (%)	24.3
Bifurcation (%)	21.4
Stent length (mean ± SD) (mm)	21.4 ± 8.2
Post-dilatation (%)	75.7
Overlapping (%)	2.9
%DS < 30 after PCI (visually) (%)	98.6

^a^Moderate + severe

The mean lesion length was 14.64 ± 7.58 mm, reference vessel diameter was 1.95 ± 0.28 mm, and the post-procedural in-stent MLD was 1.99 ± 0.27 mm. All stents were successfully implanted in all patients (Table 3).

**Table 3:** Baseline and follow-up angiographic data

Number of patients	70
Number of lesions	70
Before procedure	
Lesion length (mm)	14.64 ± 7.58
Reference vessel diameter (mm)	1.95 ± 0.28
Minimal lumen diameter (mm)	0.67 ± 0.23
Diameter stenosis (%)	65.5 ± 9.9

	In stent	In segment
After procedure		
Minimal lumen diameter (mm)	1.99 ± 0.27	1.57 ± 0.38
Diameter stenosis (%)	11.7 ± 8.70	29.7 ± 11.7
Acute gain (mm)	1.31 ± 0.29	0.89 ± 0.36

### Angiographic outcomes

Nine-month follow-up angiography was performed in 69 patients (98.6%). The MLD was 1.76 ± 0.35 mm, indicating that sufficient vessel diameter was maintained. In particular, the proximal edge showed good patency with an MLD of 1.93 ± 0.44 mm. In-stent and in-segment LLL at 9 months were 0.22 ± 0.31 and − 0.02 ± 0.34 mm, respectively (Table 4).

**Table 4:** Angiographic outcomes at 9 months

Number of lesions	69
Minimal lumen diameter (mm)	
In stent	1.76 ± 0.35
In segment	1.59 ± 0.39
Diameter stenosis (%)	
In stent	19.6 ± 13.6
In segment	27.2 ± 13.6
Binary restenosis rate (%)	
In stent	4.3
In segment	7.2
Late lumen loss (mm)	
In stent	0.22 ± 0.31
In segment	− 0.02 ± 0.34

### Clinical outcomes

**Fig. 1:**
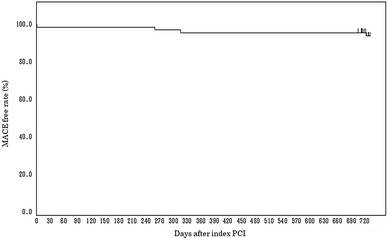
Kaplan–Meier curve of MACE-free rate up to 2 years after implantation of Ultimaster φ2.25 mm stent

Major adverse cardiac events were observed in 2 patients (2.9%) at 9 months after the procedure, giving an MACE-free rate (primary endpoint) of 97.1% (Table 5). The lower limit of the two-sided 95% CI of the MACE-free rate at 9 months was 90.1%, which exceeded the lower limit for demonstrating superiority to POBA (80%), indicating better outcomes with the 2.25-mm diameter Ultimaster stent compared to POBA. The breakdown of MACE was non-Q MI in one patient and clinically driven TLR in the other. TLF and TVF were observed in 2 patients (2.9%) and 3 patients (4.3%), respectively. Between 9-month and 2-year two TLRs were recorded. MACE rate was 8.6% up to 2-year (Fig. 1). There were no instances of stent thrombosis (as defined by the Academic Research Consortium): bleeding and vascular complication (as defined by the Bleeding Academic Research Consortium) through 2 years. There was no device malfunction, such as stent crossing failure or fracture (Table 6).

**Table 5:** Primary outcomes at 9 months

	Patients	Event free	Point of estimate (%)	95% CI (2-sided)
MACE-free rate	70	68	97.1	90.1–99.7

*MACE* cardiac death, a composite of cardiac death, target vessel myocardial infarction (MI), and clinically driven target lesion revascularization (TLR)

**Table 6:** Clinical outcomes at 9 months and at 2 years

	9 months (*N* = 70 patients)	2 years (*N* = 70 patients)
Death	0 (0%)	0 (0%)
Any cause	0 (0%)	0 (0%)
Cardiac	0 (0%)	0 (0%)
MI	1 (1.4%)	3 (4.3%)
Q MI	0 (0%)	0 (0%)
Non-Q MI	1 (1.4%)	3 (4.3%)
Clinically driven TLR	1 (1.4%)	3 (4.3%)
Clinically driven TVR	2 (2.9%)	5 (7.1%)
MACE	2 (2.9%)	6 (8.6%)
Death	0 (0%)	0 (0%)
Q MI	0 (0%)	0 (0%)
Non-Q MI	1 (1.4%)	3 (4.3%)
Clinically driven TLR	1 (1.4%)	3 (4.3%)
TLF	2 (2.9%)	4 (5.7%)
TVF	3 (4.3%)	6 (8.6%)
Stent Thrombosis	0 (0%)	0 (0%)
Bleeding	0 (0%)	0 (0%)
Stent fracture	0 (0%)	0 (0%)

*TLF* cardiac death that cannot be clearly attributed to a vessel other than the target vessel, target vessel MI, and clinically driven target lesion revascularization, *TVF* cardiac death that cannot be clearly attributed to a vessel other than the target vessel, target vessel MI, and clinically driven target vessel revascularization

## Discussion

The main findings of our study are that 2.25-mm Ultimaster DES performed very well in this challenging patient and lesion characteristics setting despite being a more difficult condition (higher age, higher rate of patients with diabetes, smaller reference vessel diameter, and longer lesion) than the meta-analysis used for setting performance goal. The rate of MACE was very low, with no stent thrombosis nor bleeding observed through 2 years.

Our data add further evidence to the performance of this new stent with bioresorbable abluminal gradient coating and reduced dose of antiproliferative drug.

### Differences in clinical outcomes among different vessel diameters

van der Heijiden et al. reported 2-year outcomes for the DUTCH PEERS (TWENTE II) trial conducted using Resolute and Promus everolimus-eluting stents (EESs). It was shown that MACE and TLF were significantly more frequent in vessels < 2.5 mm in diameter than in larger vessels [MACE: 10.8 vs. 6.8% (*P*_adj_ = 0.03), TLF: 9.5 vs. 5.4% (*P*_adj_ = 0.02)] [12]. More detailed analysis revealed no significant differences between vessels of < 2.25 vs. 2.25 to < 2.5-mm, and between those of 2.5 to < 2.75 vs. ≥ 3.0-mm in diameter. Therefore, a vessel diameter < 2.5-mm may be regarded as a threshold for predicting clinical outcomes. The results of this study are particularly relevant, because it is an all-comer study and as such, representative of daily practice. These results show that even when DES are used, treatment of very small vessels can still be challenging depending on patient and lesion-specific characteristics.

Similar to Heijiden et al., Wöhrle et al. reported the clinical outcomes for two stent types, wherein the diameter of small vessels was also subdivided into three categories < 2.0, 2.0–2.25, and 2.25–2.5 mm [13]. When clinical outcomes were evaluated for Ultimaster SES and Xience EES, no difference between the two stents was found. The event rate in the study for both stents was in line with Heijiden et al., but higher than in our current study. CENTURY II was also a study with very limited exclusion criteria, and therefore, when interpreting results of the current single-arm study, it is important to consider differences in patient characteristics.

**Fig. 2:**
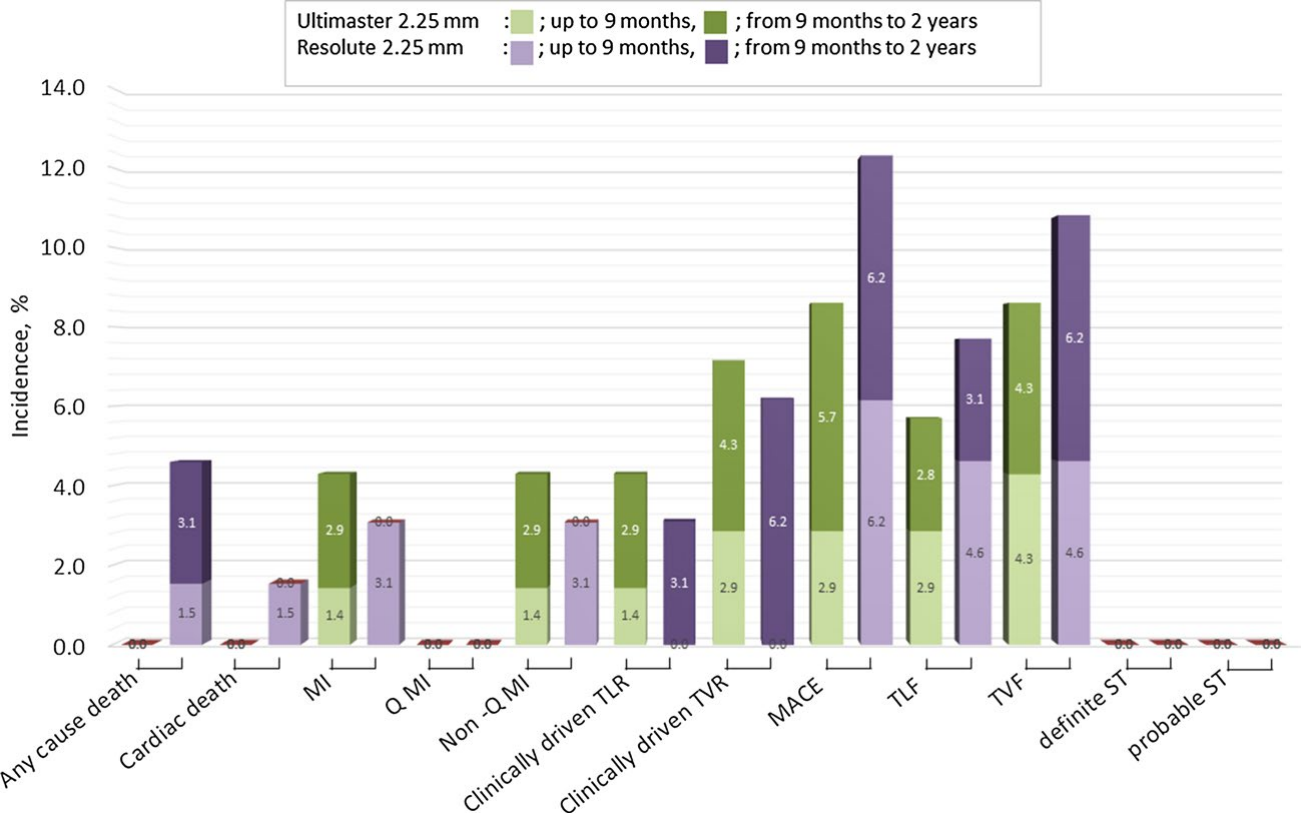
Comparison of clinical outcomes at 9 months and 2 years with RESOLUTE φ2.25 mm

Yeung et al. reported the outcomes for the subset of patients treated with 2.25-mm Resolute stent from the RESOLUTE US trial [10]. The 12-month TLF rate was 4.8%. Similar to our study, Saito et al. [8] reported results of the RESOLUTE Small Vessel Study that evaluated Resolute in Japanese patients (RESOLUTE Japan SV study). There were some differences of patient and lesion characteristics between CENTURY JSV study and RESOLUTE Japan SV study. Rate of IDDM was significantly higher in RESOLUTE Japan SV study (10.8 vs. 0%); in the other hand, ratio of B2 and C in lesion classification was higher in CENTURY JSV study (68.6 vs. 45.1%). There is no difference in other characteristics (Tables 7, 8). The MACE rate through 2 years was 12.3% which was similar to our results (8.6%), and importantly, in both studies, TLR rate was exceptionally low (Fig. 2). Although the stenosis and even occlusion of small vessels would cause less angina complaints, due to the small portion of myocardium supplied by those vessels and as such, less frequently require repeat revascularization, in the case of angiographic follow-up, the rate of revascularization is shown to be higher. In the current study, there was two (2.9%) TLRs between 9 months and 2 years, further confirming excellent antiproliferative feature of those new generation DES.

**Table 7:** Patient demographics of CENTURY JSV and RESOLUTE Japan SV

	CENTURY JSV (*N* = 70 patients)	RESOLUTE Japan SV (*N* = 65 patients)	*P*
Age (mean ± SD)	70.4 ± 9.2	69.4 ± 9.5	–
95% confidence interval	68.2–72.6	67.0–71.8	
Gender, male (%)	77.1	67.7	0.30
Type of angina (%)			–
Stable	87.1	–	
Unstable	5.7	–	
Silent ischemia	7.1	–	
Diabetes (%)	37.1	41.5	0.73
IDDM (%)	0	10.8	0.015
Hypertension (%)	87.1	87.7	1.0
Dyslipidemia (%)	87.1	80	0.37
Cerebrovascular disease (%)	2.9	–	–
Peripheral artery disease (%)	7.1	–	–
Congestive heart failure (%)	4.3	–	–
Family history of CAD (%)	27.1	–	–
Current smoker (%)	11.4	9.2	0.89
Previous PCI (%)	52.9	67.7	0.11
Previous CABG (%)	0	–	–
Previous MI (%)	27.1	32.3	0.64
Previous Stroke (%)	11.4	13.8	0.87

*P* value; *χ*^2^ test

**Table 8:** Lesion characteristics of CENTURY JSV and RESOLUTE Japan SV

	CENTURY JSV (*N* = 70 patients)	RESOLUTE Japan SV (*N* = 65 patients)	*P*
Number of lesions	70	71	
Target vessel location (%)			0.40
LMT	0	–	
LAD	32.9	35.4^a^	
LCx	42.9	35.4^a^	
RCA	24.3	38.5^a^	
Lesion classification (%)			–
A	8.6	–	
B1	22.9	–	
B2	35.7	45.1	0.008
C	32.9		
Bend (> 45°) (%)	8.6	–	–
Calcification^b^ (%)	7.1	–	–
Tortuosity^b^ (%)	24.3	–	–
Bifurcation (%)	21.4	–	–
Stent length (mean ± SD) (mm)	21.4 ± 8.2	19.25 ± 5.98	
95% Confidence interval	19.4–23.4	17.84–20.67	
Post-dilatation (%)	75.7	73	0.89
Overlapping (%)	2.9	–	–

*P* value; *χ*^2^ test

^a^Percentage to the total number of patients

^b^Moderate + severe

**Fig. 3:**
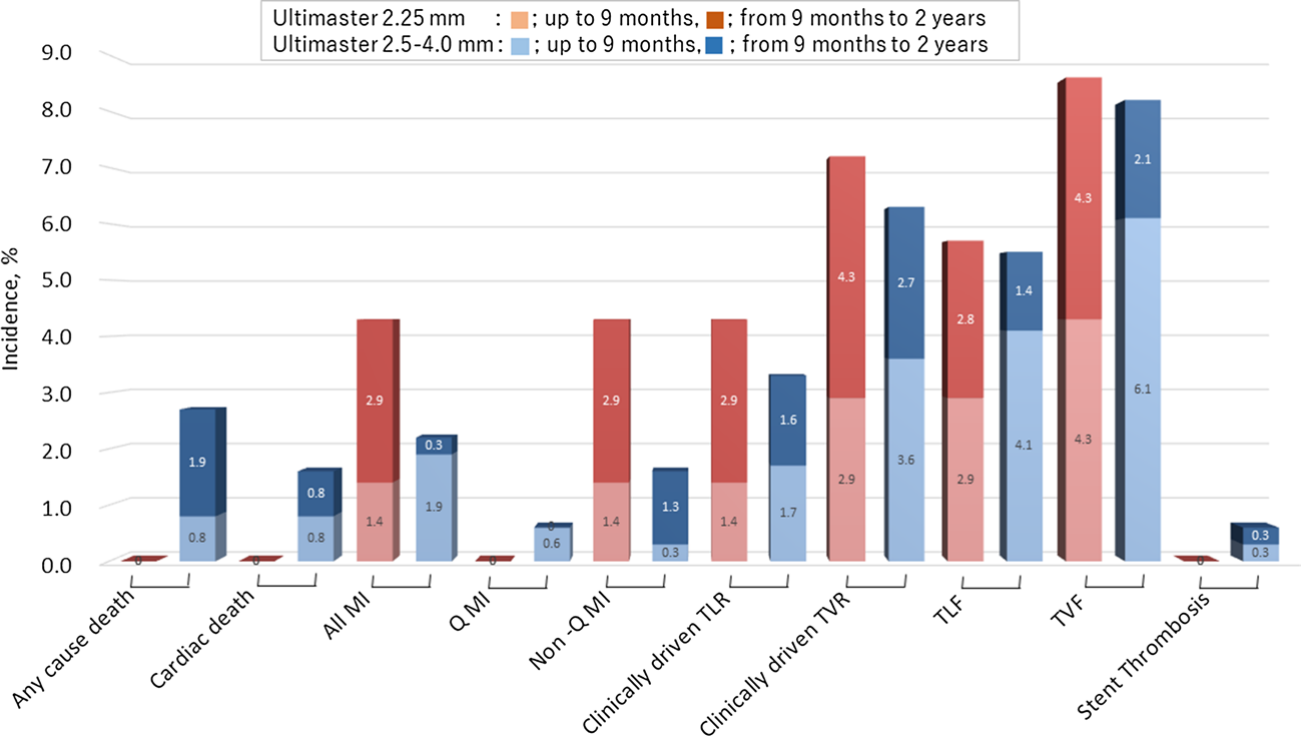
Comparison of clinical outcomes at 9 months and 2 years with other sizes of Ultimaster DES

To verify whether the clinical outcomes of Ultimaster are affected by the stent diameter, we compared the results from the present study with those of the CENTURY II study, a pivotal study of Ultimaster stents that used stents with a diameter of 2.5–4.0 mm [14]. The CENTURY II study included an all-comer cohort (cohort B) and a cohort with more restrictive inclusion criteria compliant with Japanese regulations (cohort JR). Since inclusion criteria in CENTURY JSV trial were more similar to cohort JR, we compared our findings with the outcomes of this population. Despite some differences in overall risk factors in the two studies, the clinical outcomes were similar, with TLF rates in current study of 5.7% and in CENTURY II 5.5% (Fig. 3). This finding reassures stable performance of Ultimaster DES across the wide patients and lesions characteristics, vessel size, as well as geographic areas. Thin struts, high flexibility, and conformability to the vessel wall of Ultimaster DES, along with bioresorbable polymer are features that alone or in synchrony could contribute to those promising findings.

### Long-term benefits of bioresorbable polymer

Several studies have reported the outcomes of (first or second generation) DESs in small vessels. Most of these studies were conducted using durable-polymer DESs, and the reported MACE rates at 2 years were about 10–20% for first-generation DESs and about 10% for second-generation DESs. Although these outcomes were largely satisfactory, over a prolonged period, the remaining polymer has been shown to induce inflammation associated with excessive coronary artery contractions and the development of vasa vasorum. It has been suggested that these long-term events may be preventable using a bioresorbable-polymer DES, which eventually becomes a BMS, because inflammation due to the polymer is reduced after the polymer disappears [15, 16]. Regarding outcomes for bioresorbable-polymer DESs, Wöhrle et al. have reported the results of vessels ≤ 2.5 mm in diameter as a subgroup analysis of the CENTURY II trial. These comprised an RCT of bioresorbable-polymer DES (Ultimaster) and durable-polymer DES (Xience) [13]. They found that the 12-month TLF was similar between the bioresorbable-polymer DES (6.9%) and durable-polymer DES (7.7%).

In the present study, although in a small sample size, the incidence of MACE during the initial 9 months was low at 2.9%, and the angiographic in-stent late lumen loss was also low (0.22 mm). This effect may be due to the polymer resorption and drug release time being both 3–4 months. As such drug can suppress inflammation potentially caused by polymer degradation, leaving only thin bare metal stent embedded into newly formed intima, as shown in serial optical frequency domain imaging [17]. However, to clarify the benefit of bioresorbable-polymer DES for the treatment of very small vessels, further examination of long-term outcomes is necessary.

### Study limitations

Although strictly monitored and well controlled, CENTURY JSV is a small single-arm study. Consequently, it is difficult to make comparisons with other stents that can only be done in the randomized studies.

The reported follow-up period was relatively short not allowing to fully explore potential benefit of bioresorbable polymer; however, follow-up of the enrolled patients in this study will continue until 5-year allowing the assessment of long-term outcomes.

Lesions treated were small diameter, so ischemic symptom might be relatively weak. It may have overestimated the performance of this stent.

As this study enrolled Japanese patients only, routine procedures and medications may be different from those used in other regions.

## Conclusions

From 2-year result of CENTURY JSV study, the 2.25-mm diameter Ultimaster^®^ bioresorbable-polymer sirolimus-eluting stent was found be safe and effective for the treatment of lesions in very small coronary arteries in Japanese population.

To confirm the effectiveness of the bioresorbable polymer compared with a durable-polymer DES, it is necessary to obtain the long-term results from a randomized controlled trial.
